# Cognitive Service Virtualisation: A New Machine Learning-Based Virtualisation to Generate Numeric Values

**DOI:** 10.3390/s20195664

**Published:** 2020-10-03

**Authors:** Zeinab Farahmandpour, Mehdi Seyedmahmoudian, Alex Stojcevski, Irene Moser, Jean-Guy Schneider

**Affiliations:** 1 School of Software and Electrical Engineering, Faculty of Science, Engineering and Technology, Swinburne University of Technology, Melbourne, VIC 3122, Australia; mseyedmahmoudian@swin.edu.au (M.S.); astojcevski@swin.edu.au (A.S.); imoser@swin.edu.au (I.M.); 2 School of Information Technology, Faculty of Science Engineering and Built Environment, Deakin University, Geelong, VIC 3220, Australia; jeanguy.schneider@deakin.edu.au

**Keywords:** service virtualisation, machine learning, cognitive system, quality assurance

## Abstract

Continuous delivery has gained increased popularity in industry as a development approach to develop, test, and deploy enhancements to software components in short development cycles. In order for continuous delivery to be effectively adopted, the services that a component depends upon must be readily available to software engineers in order to systematically apply quality assurance techniques. However, this may not always be possible as (i) these requisite services may have limited access and (ii) defects that are introduced in a component under development may cause ripple effects in real deployment environments. Service virtualisation (SV) has been introduced as an approach to address these challenges, but existing approaches to SV still fall short of delivering the required accuracy and/or ease-of-use to virtualise services for adoption in continuous delivery. In this work, we propose a novel machine learning based approach to predict numeric fields in virtualised responses, extending existing research that has provided a way to produce values for categorical fields. The SV approach introduced here uses machine learning techniques to derive values of numeric fields that are based on a variable number of pertinent historic messages. Our empirical evaluation demonstrates that the Cognitive SV approach can produce responses with the appropriate fields and accurately predict values of numeric fields across three data sets, some of them based on stateful protocols.

## 1. Introduction

Enterprise software systems are increasingly structured as a number of distributed components, called services, which communicate with one another. Individual services of an enterprise system often communicate with external requisite services from many different vendors to accomplish their tasks [1]. However, a number of factors may prevent software development teams from developing components for enterprise systems on time, within budget, and with the expected quality and performance. Such a phenomenon occurs because the services that a newly developed (or significantly modified) component depends upon may be accessible for a limited time only or at a significant (monetary) cost. This situation may result in new components only being thoroughly tested after the completion of the development, thereby putting an entire deployment environment at risk if any undetected defaults cause ripple effects across multiple services in the respective deployment environment.

**Figure 1:**
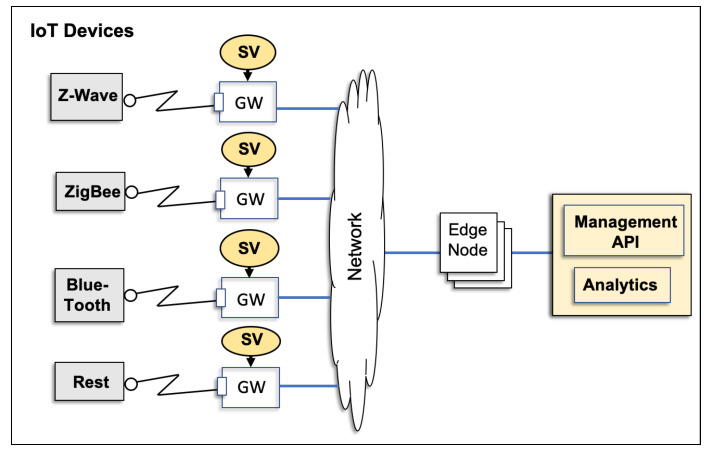
IoT nodes and their communication.

This phenomenon will be significantly highlighted in IoT applications, such as sensor recorded data where the data model places a vital role in the performance of the relevant systems [2]. IoT services need to communicate with many IoT devices that may be sensors, actuators, or gateways (see Figure 1).

Continuous delivery and testing of IoT services are particularly challenging because of the heterogeneous hardware, multiple communication layers, lack of industry standards, and skill sets requiring OT and IT. These challenges can delay the release of the application and affect software quality. Releasing the IoT application may require the physical devices to be present every time that the application is fully tested. IoT virtualisation can remove constraints for IoT development. A virtual test-bed of IoT devices can accelerate the IoT application development by enabling automated testing without requiring a continuous connection to the physical devices.

The IoT concept has initiated the smart cities, as presented by Bhagya, et al. [3]. The aim in the smart cities is to improve the citizen’s quality of lives by intelligently supporting the city functions with minimal human interplay. However, testing the integration of multiple smart components each consists of innumerable heterogeneous devices, sensors, several communication models, and protocols could be one of the most challenging tasks when deploying a real world smart city [3].

Continuous delivery is an industry best-practice for accelerating software delivery and increasing software quality [4]. This practice has been introduced in order to mitigate the challenges of significant integration costs when simultaneous changes to components and their requisite services are irregularly synchronized (and “mismatches” quickly fixed). To effectively adopt continuous delivery, requisite services must be readily available to the software engineers of a development team; otherwise, continuous integration and testing cannot be efficiently performed.

A number of techniques have been proposed for providing ‘replicated’ services in development and testing environments in order to ensure that they can be readily accessed by software engineers. Mock objects and stubs [5,6] are widely used to mimic the interactive behaviour of actual server-side systems. However, this approach is language-specific, and any modification in the systems leads to a change in the code. Another solution uses virtual machines or hardware virtualisation tools, such as VMWare and VirtualBox [7], to host multiple server-side systems that communicate with a component under development. This method assumes the availability of the real server systems for installation. These systems are often large and they do not scale well to large numbers of deployments. Container technologies, such as Docker [8], which virtualise operating system environments rather than hardware and make available protected portions of the operating systems, are also used for setting up testing environments. Container technologies are lightweight and provide good scalability; however, they mostly suffer from the same limitations as hardware virtualisation approaches.

Recently, service emulation [9,10] and service virtualisation (SV) [11] have been introduced. Both approaches aim to replace any requisite services for a component under development with lightweight, executable “approximations” thereof that can be easily configured and deployed without replicating the requisite services and/or their deployment environments. The aforementioned approaches also aim to only replicate real services’ behaviour for specific quality assurance tasks, but they mostly ignore any other features or properties of the real services.

In service emulation, the executable model of a requisite service has to be manually specified [9,10]. This task requires significant knowledge regarding the functionality of a requisite service, which is something that may not always be readily available (e.g., in case of legacy software) and practical when a requisite service’s behaviour is quite complex. Some shortcomings of service emulation are addressed in SV by (i) recording the interactions between a component under development and its requisite services (e.g., using tools, such as Wireshark [12]), from a number of test scenarios and (ii) generating executable models of the requisite services by applying data mining techniques [6,11,13,14]. Consequently, some of these techniques are referred to as “record-and-replay” techniques. For example, the technique that was presented by Du et al. [14] “matches” an incoming request with those found in the aforementioned recordings and substitutes fields to generate a response for the incoming request. This technique was refined and it became sophisticated over the years (Versteeg et al. [15]).

One of the key drawbacks of these SV approaches is that they only focus on stateless protocols—the response of a service only depends on the current request, but not on any prior interactions—and hence fail to adequately work for protocols that have inherent side-effects on the internal state of a service (as found in state-full protocols). Enişer and Sen [16] employed classification-based and sequence-to-sequence-based machine learning algorithms to cater for state-full protocols. However, their work can only project values of a categorical type, thereby failing to address the accurate projection of numeric values, amongst others.

This paper presents a novel software virtualisation solution, called Cognitive Service Virtualisation (CSV), which is capable of projecting numeric values in response to service requests. This project employs a new methodical approach for the first time in this field. The proposed approach uses data mining methods and time series analysis concept to find the relationship between each numeric field in the service response with their previously sent/received request/response messages to achieve an accurate model for each numeric field in the service response.

The rest of this paper is organised, as follows: in Section 2, a number of key related studies are discussed. The problem to address in this work is formalised in Section 3, followed by a motivating scenario in Section 4, which illustrates the challenge of predicting numeric fields in response messages. Section 5 presents the key steps of the proposed CSV approach. The data sets used for our evaluation and the results of the evaluation are introduced in Section 6 and Section 7, respectively. Section 8 discusses threats to validity to our evaluation. The paper concludes in Section 9 with a summary of the main observations and future work.

## 2. Related Work

Mining service behaviour has become a topic of interest in many fields of software engineering; accordingly, much research in recent years has focused on the development of methods for addressing it. SV is one use case of service behaviour mining that focuses on the discovery of control and data aspects of software services from their network interactions. Researchers have examined and improved many aspects of SV.

Kaluta [17] is a specification-based service emulation tool that replaces different types of requisite systems with the execution of a runtime behaviour model on the basis of the protocol specifications of their expected interactions with SUT. Kaluta provides a framework that can model the behaviour of services in a compact and convenient way. This framework uses the description of the services’ protocols, behaviour and data models, and provides a runtime emulation of the service in a testing environment for responding to requests from the system under test, thereby reportedly achieving a high scalability.

In recent years, commercial enterprises have developed service virtualisation tools. Computer Associates, [18], IBM [19], and HP [20] strive to speed up software development by using virtualisation tools. They use service and protocol specifications along with inferred information from the traces to create a virtual service model.

Opaque SV [15] has been introduced in order to virtualise stateless services. This method receives a raw message as input and generates its response by using clustering and sequence alignment techniques without any prior knowledge of the protocol message format. The aforementioned method addresses encoding and decoding of the protocol and generating the response message with the correct content. This method works well when a simple equivalence dependency exists between each request and its corresponding response. However, this method cannot accommodate many complex dependencies between the request and corresponding response in stateless services or protocols.

Enişer and Sen [16,21] proposed three different ways of generating responses to a request in order to address stateful services. In the first method, they used a classification that maps each request and its previous messages to a class of possible values for each field in the response message. They considered the fields’ values as categorical data and employed a classifier to map the request and historic messages to one of the categories in each field in the response message. The second method that they proposed is sequence-to-sequence based virtualisation using a special RNN structure, which consists of two LSTM networks. LSTMs have been found to be efficient with sequence-based data sets that contain categorical or textual data in language translation use case [22]. The third method uses a Model Inference Technique (MINT) [23] to generate each categorical response field. MINT is a tool for inferring deterministic and flexible Guarded Extended Finite State Machines (EFSM) that use arbitrary data-classifiers to infer data guards in order to generate the guard variables and merge the states. The training time for RNN structure is long, thereby leading to the implementation of the method on GPU. All models with the proposed structures were reported to work well with protocols that have textual and categorical data fields. However, these models cannot accommodate non-categorical numeric fields.

Existing service emulation and virtualisation approaches have major limitations. First, developing a service specification in Kaluta requires significant developer effort, and its configuration and maintenance are complex. Second, an opaque SV is stateless and it generates a response to an incoming request only on the basis of the simple dependency to its corresponding request without considering the interaction history, thereby resulting in the inaccuracy of complicated protocols. Third, although the latest published work [16] can accommodate stateful services, it cannot generate a response message that contains accurate non-categorical numeric data. In addition, there are limits regarding how long the LSTM-based method can take the recorded messages into account. LSTM has a sequential structure and it uses back-propagation to train the model; therefore, it takes a long time on a normal system to be trained [24].

## 3. Problem Definition

Software services communicate with each other by exchanging messages, often with the premise that one service sends a request to another service expecting to receive a response (or possibly several responses) to the request within a certain amount of time. The types of messages that are exchanged often follow the rules defined by an underlying protocol. Specifically, these types must adhere to the (i) specific structures on how information is encoded and transmitted over a network and (ii) temporal dependencies that determine in which order certain messages can be exchanged.

In this work, we assume that each request message emitted by a service will always result in a single response. In situations where a protocol does not strictly follow the notion of request/response pairs (e.g., LDAP [25] search requests may result in multiple responses), we apply the technique that was proposed by Du et al. [14] to convert exchanged messages into request/response pairs.

Different protocols apply varying techniques in order to structure the information to be transmitted and how the resulting structure is encoded into a format suitable for effective communication over a computer network: some protocols use a textual encoding for transmission (e.g., SOAP), whilst others use a binary encoding (e.g., LDAP).

Herein, we assume that we have a suitable encoder/decoder that can read messages sent in a protocol’s “native” format and convert them to a non-empty sequence of key/value pairs with unique keys, and vice versa. If a protocol allows for the repeated use of a key across messages (for example, in LDAP [26]), then a suitable strategy is applied to make keys unique within a message (e.g., adding a number pre- or postfix to each repeated key).

**Figure 2:**
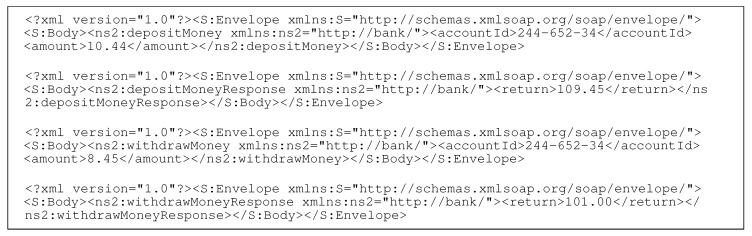
Two request and response messages of a SOAP banking data set.

Finally, we assume that we can identify a specific key across all request messages of a protocol that defines the request type (or operation type) of a given request (e.g., depositMoney or withdrawMoney; Figure 2). If the key for the request type cannot be readily identified, then we can use the technique proposed by Hossain et al. [27] to generate such a key. A domain expert may also manually apply annotations. Response messages may or may not have an equivalent response type indicator. However, the proposed approach does not require the presence of explicit response types.

Given these assumptions, we define a number of constructs needed to formally express our approach. First, we have the set of keys, denoted by K, and require that equality and inequality be defined for the elements of K. We use *k* and $k_{i}$ to denote elements of K.

Furthermore, we have the set of values, denoted by V, and require that equality and inequality be defined for values. We use *v* and $v_{j}$ to denote the elements of V. Message *m* is a non-empty sequence of key/value pair bindings with each key k∈K to appear at most once in *m*. We use *m* and $m_{k}$ to denote the messages and the set of all messages as M and use the notation

$$m\;=\;<k_{1},v_{1}><k_{2},v_{2}>\ldots <k_{n},v_{n}>$$

to denote a message with keys $k_{1},k_{2},\ldots k_{n}$ and the corresponding values $v_{1},v_{2},\ldots v_{n}$. We write $K_{m}$ to denote the set of keys that are used in a message *m*. In the example above, $K_{m}=\left\{k_{1},k_{2},\ldots ,k_{n}\right\}$.

A specific key/value pair <$k_{p}$, $v_{p}$> is often referred to as field, and we use the terminology that a message is defined by a non-empty sequence of fields. We also use the notation $m_{q}$ and $m_{p}$ to explicitly denote the request and response messages, respectively, if this is not immediately obvious from the context.

Consider the four XML-encoded messages of a banking service that are given in Figure 2: the first message defines a depositMoney request; the second message is the corresponding response; the third message represents a withdrawMoney request; and, the fourth and final messages are the corresponding responses. DepositMoney and withdrawMoney denote two of the six possible request types of the corresponding protocol.

We encode the following four messages into a non-empty sequence of fields (i.e., key/value pairs):

$$\begin{array}{cc} m_{q_{1}}= & <\mathrm{NS}2,\mathrm{depositMoney}><\mathrm{accountID},244-652-34><\mathrm{amount},10.44>\\ m_{p_{1}}= & <\mathrm{NS}2,\mathrm{depositMoneyResponse}><\mathrm{return},109.45>\\ m_{q_{2}}= & <\mathrm{NS}2,\mathrm{withdrawMoney}><\mathrm{accountID},244-652-34><\mathrm{amount},8.45>\\ m_{p_{2}}= & <\mathrm{NS}2,\mathrm{withdrawMoneyResponse}><\mathrm{return},101.00> \end{array}$$

The value of the key ‘NS2’ encodes the request type of both request messages, whereas the values of the other two keys denote information of the corresponding requests.

In this work, we denote a request message and its corresponding response as an Interaction. In the banking service example, $m_{q_{1}}$ and $m_{p_{1}}$ denote one interaction and $m_{q_{2}}$ and $m_{p_{2}}$ as another interaction. We use I to denote the set of interactions (*I*, $I_{i}$ etc.) to denote individual iterations. Finally, we denote non-empty sequences of interactions $I_{1}$, $I_{2}$, …$I_{j}$ as trace *T*.

We define the stateless protocol as interactions that the response message values can be generated by incorporating its request message. In the stateful protocol, a service for generating some response values may need its current request and the previous relevant interactions. The number of relevant interaction to generate a response field can vary from service-to-service, message type-to-message type, and field-to-field. If we show the number of relevant messages for a response field $F_{l}$ with $M_{l}$, then we can use $M_{l}=1$ and $M_{l}>=1$ for the stateless and stateful protocols, respectively.

A categorical variable is a variable that can be assigned one of a limited number of possible values. For convenience in processing categorical variables, they may be assigned numeric indices, for example, for k categorical variables they may assign 1 to *K*. These numbers which assigned to categorical variables are arbitrary and provide only a label for each specific value. These categorical labels follow different logical concepts than the assigned numbers, therefore, cannot be manipulated as numbers. Instead, valid operations are equivalence, set membership, and different distribution related operations, such as joint probabilities, etc. As the result, we consider that categorical fields do not need mathematical calculations to be generated; therefore, we identify the numeric fields that can be considered as categorical and remove them from the list of numeric fields.

Given these preliminaries, we can now formally define the problem of service virtualisation, as follows: given a sequence of interactions $I_{0}$, $I_{1}$, …$I_{j-1}$ and a request message $m_{q_{j}}$, a virtual service is to generate a response $m_{p_{j}}$ that resembles as closely as possible the response a real service would create; specifically, it only contains the complete set of keys the real service would create with the values expected from the real service.

We can split the service virtualisation problem into four sub-problems:

- What keys does a virtual service need to generate in the response message?
- What values does it need to generate for each of these keys?
- Which messages are relevant to generate the specific fields’ values?
- How many relevant messages are required to generate these values?

We will address these four sub-problems in the following sections with special focus on generating the values for numeric fields.

Given that the proposed solution uses machine learning models, any machine learning classifiers can be added to the collection of the functions to analyse the data; therefore, CSV’s ability can be constrained to the limitations of the machine learning models in identifying the relationships between data. In this study, we consider the numeric modules to discover the linear and nonlinear relationships between numeric fields, such as multiplication and exponential, in the case of a large amount of data to predict them. However, some fields’ values, such as random fields, encrypted fields, hash fields, and tokens, use complex algorithms to prevent their regeneration. The use of machine learning algorithms to accurately generate data seems impossible and beyond the scope of this work. Therefore, we exclude the aforementioned fields from our evaluations.

For example, banking data set that contain getNewToken, deleteToken, getTransactions, getAccount, deposit, and withdraw transactions have linear relationships. The calculator data set, including the plus, minus, multiply, and division, is an example of linear and nonlinear (i.e., exponential) relationships.

## 4. Motivating Scenario

We consider the two requests (and their responses) given in Figure 2 in order to illustrate the limitations of a number of “record-and-replay” approaches to service virtualisation. The first requests deposits some money (in this case, 10.44) into an account with the ID 244-652-34, thereby resulting in a new balance of 109.45. The second request withdraws 8.45 from this account, thereby resulting in a new balance of 101.00.

We assume that these two messages are the last interactions that have been recorded for the purpose of the following discussion. Furthermore, we assume that we have an incoming request that withdraws 7.25 from the same account at playback time.

Opaque Service Virtualisation [15,28]

1. matches the incoming request with the requests of all recorded interactions. In our example, the second request message is the closest match;
2. identifies symmetric fields, which is, information that appears in the “closest match” recorded request and the response messages. In our example, no symmetric fields are present because no information is copied from a request into the corresponding response in the XML-based banking protocol; and,
3. replaces symmetric field information in the recorded response by the corresponding value in the incoming request. In our example, the recorded withdrawMoneyResponse remains unchanged due to the absence of symmetric fields.

The response message that is generated in Step 3 is now returned as the response to an incoming request. In our scenario, the withdrawMoneyResponse message given in Figure 2 will be returned unchanged. Although this response is protocol conformant (the response generated to a withdrawMoney request is a withdrawMoneyResponse as required by the underlying protocol) and well-formed (the response has the correct structure), the new balance that is returned is incorrect (i.e., 101.00 instead of 93.75). Specifically, the response generated is inaccurate with regard to some of the information it carries.

This example demonstrates the limitations of Opaque Service Virtualisation when it comes to stateful protocols (such as the banking example). Similar limitations exist when information in a response has a more complex relationships with information in the corresponding request than what symmetric fields can express (e.g., the message ID of a response is the message ID of the incoming request with an added fixed offset). In the following section, we present an approach that allows for us to address such shortcomings.

## 5. Csv Structure

At a high level, we consider CSV as a black box with the following input and output. The input and output messages consist of one or more (denoted by ${}^{+}$) key-value pairs $<key,value{>}^{+}$.

CSV does not impose any constraints on the meaning or formatting of the <key, value> pairs. Based on a history of relevant messages and the request message for which a response is sought, CSV derives a response template (RT) (i.e., the set of keys that the response is expected to contain) and develops the values of the keys that have been found to be numeric in the learning phase. Some numeric values are constants, whilst others follow a pattern that may depend on a trace of historic messages. The protocol at hand is stateless if the values that have to be inserted into the RT depend on the request message only. If the content of the response depends on a number of relevant past request and/or response messages, then the protocol is stateful. CSV can derive responses for stateless and stateful protocols. CSV derives the relevant messages and their number and the appropriate numeric model from a history of traces.

**Figure 3:**
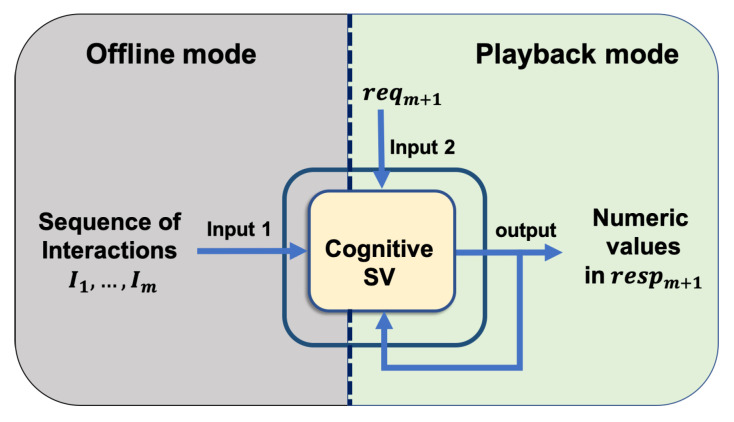
High level overview of CSV with its inputs and outputs.

The system learns from non-empty traces, which are the network interactions between SUT and requisite service. This concept is shown as Input 1 in Figure 3. Given that the historic traces contain sufficient samples of all request message types and their responses, CSV can provide a response for a new request with all required keys and accurate numeric values. The second input is the request message that requires a response whose template and numeric content CSV develops.

**Figure 4:**
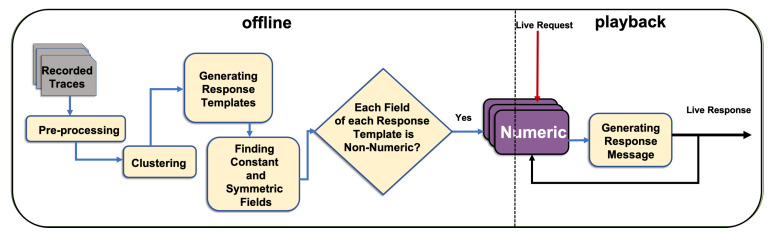
CSV structure.

**Figure 5:**
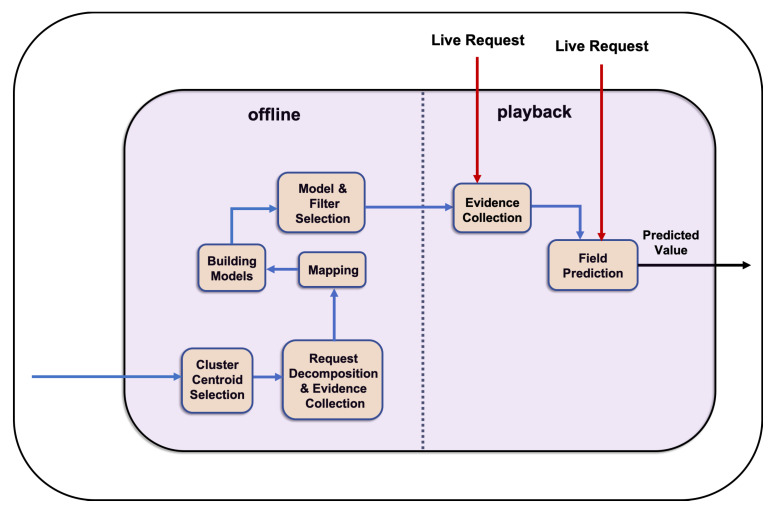
CSV’s numeric module in detail.

The learning process of CSV (Figure 4 and Figure 5) is conducted in an offline mode, which is described in detail in Section 5.1. Section 5.2 presents the application of the proposed solution to the problem of virtualising a response to a new request in playback mode.

### 5.1. Offline Mode

Figure 4 shows that the CSV in the offline phase learns a model for each numeric value in the response of each request type from the recorded interactions. The CSV also learns the appropriate template for a response to be virtualised, the constant values, the model to apply, and the number of relevant messages to derive the correct numeric values for each numeric field.

#### 5.1.1. Pre-Processing

Figure 4 presents that the pre-processing is the component in the offline learning process. This step numbers all historic messages in the order in which they were recorded. Numbering messages serves two purposes: it provides a unique ID for each message, which helps in identifying and accessing the message; and, it provides an ordering of the messages within a trace. Accordingly, each message has an additional ID field. The ID that is added as a new key to the message is not a key in the message already.

$$\begin{array}{cc} m_{q_{l}}= & <ID,101><NS2-1,depositMoney><NS2-2,depositMoney><accountID,244-652-34>\\ & <Amount,10.44>\\ m_{p_{l}}= & <ID,102><NS2-3,depositMoneyResponse><NS2-4,depositMoneyResponse><return,109.45> \end{array}$$

Pre-processing, as a last step, creates the interactions by assigning the corresponding response to each request in order to ensure that they can be considered as one entry. The interactions are ready to be processed in the following components.

#### 5.1.2. Clustering

Each request message type has its own response message. We assume that the particular set of rules that governs the generation of each field in the response to a request is defined by the type of the request message and it applies to all messages of the same type in the protocol. A prerequisite for the success of CSV is that the body of evidence—the historical traces—contains a sufficient number of message traces to discover those rules. Although the same rules may apply across the message types, the CSV learning mechanism treats each message type individually. Therefore, the interactions (request–response pairs) should be clustered by request message type.

This concept assumes that all of the interactions have a type field $<k_{q_{t}},v_{q_{t}}>$ in the request message that can be used by the clustering algorithm. In the absence of such a field, expert users can add a field to the messages or define a number of fields where the clustering algorithm can use them. As the result of clustering on the basis of the request message type, *N* clusters of interactions are generated, where *N* equals the number of distinct values of $v_{q_{t}}$ in all request messages. Each cluster contains interactions with equal request message types $v_{q_{t}}$.

$$cluster_{1}\ldots cluster_{N},N=\left|\left(distinct\left(v_{Tq}^{\prime }\right)\right)\right|$$

#### 5.1.3. Generating RTs

Each request message type has its own response with its set of keys, which makes it possible for the system to create a RT for each request type. The purpose of this component is to create the RT for each response type and then pre-fill the value of the fields that can be identified while using simple dependencies. The first step in this component is to transform all request and response messages in each cluster to a request and response common template (CT). This step is designed to assign two CTs to each cluster, one for all requests, which is denoted as $CT_{q}$, and one for all responses denoted by $CT_{p}$. In creating a $CT_{q}$:

First, all of the response messages of each cluster are collected. Subsequently, a response CT is created in each cluster, which is the union of the responses’ sets of keys in that cluster,

$$\begin{array}{cc} CT_{pC_{i}} & =k_{1},k_{2},...,k_{l}=K_{1}\cup K_{2}\cup \ldots \cup K_{h}\\ \; & \forall i\in \left\{1\ldots h\right\};\;K_{i}\subseteq K\\ \; & K=\{K_{1},K_{2},\ldots K_{h}\} \end{array}$$

The above process is repeated for all request messages in each cluster. Accordingly, a $CT_{q}$ is created for each cluster as the union of the requests’ sets of keys in that cluster.

The *N* generated $CT_{p}$ of the response keys (one $CT_{p}$ for each cluster) and *N*$CT_{q}$ of request keys, which contain different numbers *l* of keys, depending on the length of the response to each message type in that cluster for $CT_{p}$ and the length of request messages in that cluster for $CT_{q}$. Each set $K_{i}$ of a response or request type *i* is a subset of the set K of keys encountered in the protocol. $K_{i}$ in each $CT_{p}$ provides the keys for the responses to be virtualised in the playback phase.

All of the request and response messages of each cluster are transferred to their corresponding $CT_{q}$ and $CT_{p}$, respectively. Later, a RT is assigned to each cluster, which, at first, is equal to the cluster’s $CT_{p}$. Next, the keys in each RT that have null values in all the response messages of that cluster will be identified and filled with null value. In the banking example, we get to the common request and response keys that are listed in Listing 1 for each cluster by following this component.

**Listing 1:**
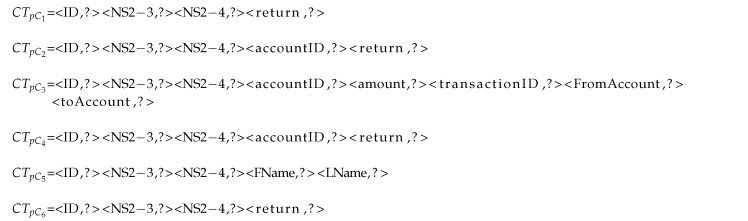
RTs for each cluster

#### 5.1.4. Finding Constant and Symmetric Fields in Each RT

We identify the constant fields in this component to exclude them from elaborate modelling in order to eliminate the need to create values for some RT fields in each cluster, which can be identified with simple methods. Three types of constants can be present in each RT. The first type is a field in RT that has the same value across all response messages of the same cluster. Fields of this type can be found by identifying response keys in each cluster for which only one non-null distinct value is present:

$$\begin{array}{cc} \forall & \;m_{p}\in cluster_{i},\exists \;<k_{p},v_{p}>,\;where\\ \; & \;v_{p}\neq Null\;\mathrm{and}\;\left|\left(distinct\left(v_{p}\right)\right)\right|=1 \end{array}$$

These fields can be removed from the cluster’s RT unknown fields to avoid further processing, because their values can be easily filled with a constant value. Accordingly, the keys with constant values are identified on the basis of the number of distinct values of each key in the same cluster. After the keys with constant values are found, we obtain the following common RTs for each cluster shown in Listing 2. In each cluster, the fields with constant values are shown in italic.

**Listing 2:**
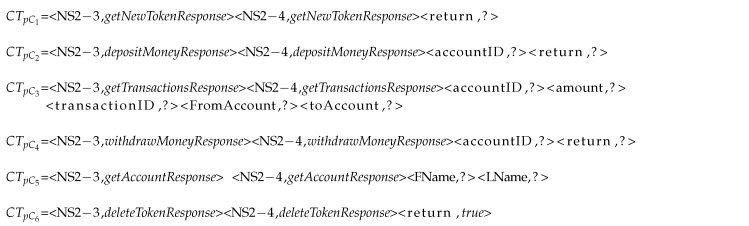
Finding keys with constant values in each cluster’s RT

In the second case, the value of a field in RT is defined by a corresponding field, of a different key, in the same RT. The number of unknown fields in each RT can be reduced by identifying this type of dependencies.

Constant fields can be identified by searching for keys in each cluster whose values in the same response are non-null and always equal:

$$\begin{array}{cc} \forall & \;m_{p}\in cluster_{i},\exists \;<k_{p_{i}},v_{p_{i}}>,\;<k_{p_{j}},v_{p_{j}}>,\;where\\ \; & \;v_{p_{i}},v_{p_{j}}\neq Null\;k_{p_{i}}\neq k_{p_{j}}\;\mathrm{and}\;v_{p_{i}}=v_{p_{j}} \end{array}$$

The third case describes a case in each cluster where fields, with the same or different keys, are present in the request and corresponding response, whose values are always equal. These fields can be removed from the RT’s unknown fields, because their values can be filled with the value of the corresponding request keys upon receiving the request message:

$$\begin{array}{cc} \forall \;m_{q} & ,m_{p}\in I,\;\forall \;I\in cluster_{i},\exists \;<k_{q_{i}},v_{q_{i}}>,\;<k_{p_{j}},v_{p_{j}}>,\\ \; & where\;v_{q_{i}},v_{p_{j}}\neq Null\;\mathrm{and}\;v_{p_{j}}=v_{q_{i}} \end{array}$$

Removing fields with null values and constants reduces the number of fields whose values have to be modelled using sophisticated algorithms. In the next step, symmetric fields that have the same values in request and corresponding response of each cluster’s template will be identified.

The result is shown in Listing 3. The fields with symmetric values are shown with the symmetric value in them that are equal to a field in their requests.

**Listing 3:**
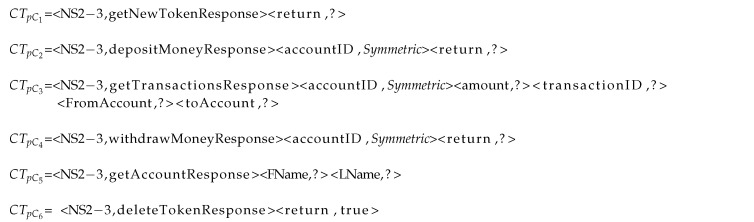
Finding keys with symmetric values

Because we address the problem of finding accurate responses for numeric fields, the non-numeric fields of RT at this point also have to be discarded. Given an unknown field <k,v> in the RT of a given cluster, *k* is tagged as the key of a numeric field if the values of *k* across all responses in that cluster only have numeric values. The keys *k* in RT that are not numeric are removed from the set of fields to be further processed.

The remaining numeric fields can have different dependencies on one or more values of the preceding request or interactions. A great number of different models may have to be generated. In the subsequent steps, models are created for each remaining RT field in each cluster.

#### 5.1.5. Tagging Each RT Field as Numeric or Non-Numeric

In the banking example that is shown in Listing 4, in each cluster, the fields with unknown values shown with *?* are given to be tagged as numeric or non-numeric.

**Listing 4:**
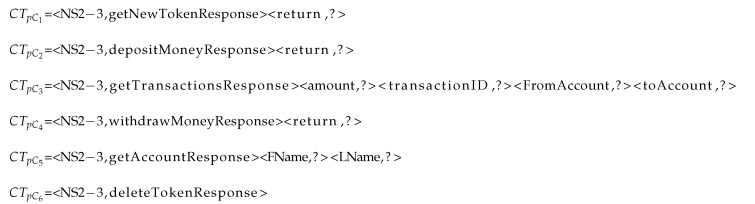
Any existing unknown fields plus message type field in each cluster’s RT

The response template in each cluster is listed, as is shown in Listing 4. $CT_{pC_{6}}$ does not have any keys with unknown values. Therefore, CSV needs to generate the values for *return* key in $CT_{pC_{1}}$, $CT_{pC_{2}}$, and $CT_{pC_{4}}$, *amount*, *transactionID*, *FromAccount*, and *toAccount* for $CT_{pC_{3}}$ and *FName* and *LName* values for $CT_{pC_{5}}$, as shown in Listing 5. However, because the value of the key return in cluster 1 is token, which as discussed before is considered as random, is excluded; therefore, in fact, only clusters 2, 3, 4, and 5 have unknown values to be generated.

**Listing 5:**
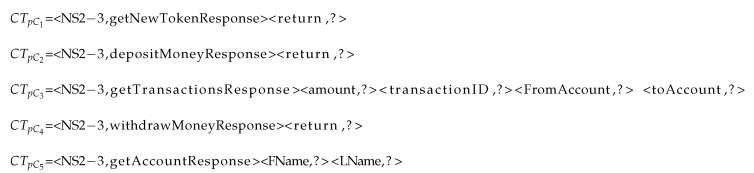
Clusters with unknown fields in their RTs

Each field can be considered to be numeric or non-numeric. Some functions in different programming languages, such as isnumeric() in Python, can be used to check whether a field is non-numeric or not. Listing 6 shows that, in each cluster, if all of the values of a field are numeric, then we consider that field as numeric and otherwise non-numeric. The isnumeric() method checks whether all of the characters of the string are numeric characters or not. The result returns True if all the characters are true; otherwise, it returns False. Numeric characters include digit characters and all the characters that have the unicode numeric value property. Numeric characters are those with the property value *Numeric Type=Digit*, *Numeric Type=Decimal*, or *Numeric Type=Numeric*.

**Listing 6:**
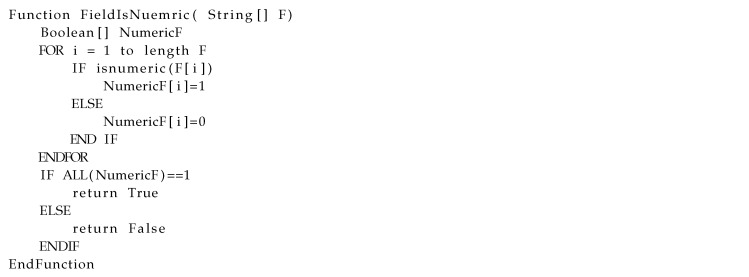
Psedu code for identifying numeric fields across each cluster

Every numeric field can be categorical or non-categorical. In case the field is recognised as numeric, it will be checked in order to determine whether it is a categorical field or not. As previously discussed, we assume that categorical fields do not need mathematical calculations to be generated; therefore, we identify the numeric fields that can be considered categorical and remove them from the list of numeric fields. The identification of the categorical type of a field is nontrivial. At present, a user is required to pre-select categorical fields. In this study, we assume that the non-categorical numeric fields are only influenced by other non-categorical numeric fields; therefore, other fields (e.g., categorical numeric and textual) are not incorporated.

#### 5.1.6. Numeric Module

The CSV tags each unknown field as numeric or non-numeric. Each unknown numeric field that is shown in Listing 7 evokes one instance of the numeric module in the offline mode. Based on the result of the component to identify the type of each unknown response fields, the *return* values of clusters 2 and 4 are identified as numeric; therefore, one instance of numeric module is separately provoked for each one of them to learn how to generate its value. The prediction of the *amount* value in cluster 3 also provokes one instance of the numeric module separately. The numeric module and its components are shown in Figure 5. For representation in this paper, we select <return,?> in $CT_{pC_{2}}$ in order to generate its values.

**Listing 7:**
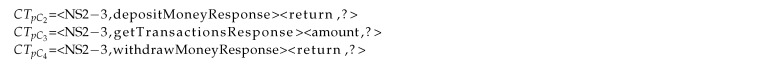
RTs of clusters with unknown numeric fields

#### 5.1.7. Cluster Centroid Selection

In each RT that has at least one unknown numeric key, we have to select a sample interaction $I_{c}=\left(\;m_{q},m_{p}\right)\;$ as a representative of its corresponding cluster to learn the more complex dependencies between each unknown numeric key in the RT and fields in the corresponding request type or other request/response types. To select the best representative message, we compare all of the request messages $m_{q}$ in each cluster. Subsequently, we select the interaction that has the request fields that are similar to the request fields of other interactions in that cluster to choose the interaction with the largest number of relevant messages in that cluster to maximise the data to learn from. We define this aspect as the centroid of the cluster. We define a distance function $\delta (\;m_{qi},m_{qj})\;$ between request messages $m_{qi}$ and $m_{qj}$, which are part of the same cluster, in order to find the centroid of each cluster:

$$\begin{array}{cc} \; & m_{q_{i}}=<k_{1},v_{i_{1}}><k_{2},v_{i_{2}}>\ldots <k_{n},v_{i_{n}}>\\ \; & m_{q_{j}}=<k_{1},v_{j_{1}}><k_{2},v_{i_{1}}>\ldots <k_{n},v_{j_{n}}> \end{array}$$

(1) $$\begin{array}{cc} \; & \delta \left(\;m_{q_{i}},m_{q_{j}}\right)\;=\sqrt[{2}]{\sum_{l=1}^{n}\left(\;w_{l}\times d\left(\;v_{i_{l}},v_{j_{l}}\right)\;\right){\;}^{2}} \end{array}$$

(2) $$\begin{array}{cc} \; & w_{l}=e\left(Base\right)-e\left(K_{l}\right) \end{array}$$

(3) $$\begin{array}{cc} \; & e\left(K\right)=-\sum_{i=1}^{s}P_{i}\times \log P_{i} \end{array}$$

(4) $$\begin{array}{cc} \; & if\;\forall i\in Field_{l},\;P_{i}=1/s\;then\;e\left(Base\right)=-\log1/s \end{array}$$

(5) $$\begin{array}{cc} & d\left(v_{i_{l}},v_{j_{l}}\right)=\left\{\begin{array}{cc} 0, & \mathrm{if}\;v_{i_{l}}==v_{j_{l}}\\ 1, & \mathrm{otherwise} \end{array}\right. \end{array}$$

The distance δ between two messages is the Euclidean distance of the weighted distances between pairs of two values $v_{i_{l}}$ and $v_{j_{l}}$ that have the same key $k_{l}$ in the $CT_{q}$ of the cluster. The weights *w* are factors that consider the importance of each field and they are calculated while using Shannon’s entropy [29]. The entropy *e* measures the amount of information that a field can carry. Given that features with great importance have less entropy, we subtract each field’s entropy $e(K_{l})$ from the base entropy e(Base) in order to reflect that the considerable important fields have great weight in the distance function. Base entropy is the entropy of a field whose values across all messages are distinct from other values, which means the highest possible entropy in each cluster.

We can define each message’s distance to all other messages in the same cluster as the sum of the distances to all of the messages in the same cluster.

(6) $$\delta \left(m_{q_{l}},C\right)=\sum_{i=1}^{s}\delta_{l,i}$$

Subsequently, we define the centroid as the interaction where the request message has the shortest distance to all other requests in the cluster:

(7) $$Centroid\left(C_{i}\right)=I_{c},where\;\min_{\begin{array}{c} \forall m_{q}\in C_{i} \end{array}}\delta \left(m_{q},C_{i}\right)=\delta \left(m_{q_{c}},C_{i}\right)$$

We select the one interaction from each cluster that has the smallest distance to all other interactions. If several interactions share the same minimal distance, then the interaction is picked with highest assigned ID from the set of interactions with minimal distance in order to maximise the chance of selecting the interaction with the largest number of history messages.

Following the banking example, each numeric module starts with cluster centroid selection. In each cluster that we have had numeric unknown response fields, the method uses Equation (7) in order to calculate the centroid interaction that has the most similar fields to the other interactions in its cluster. Based on the calculations, we have the following centroid interactions for cluster 2.

$$\begin{array}{cc} I_{c_{2}}= & <ID,101><NS2-1,depositMoney><AccountID,244-652-34><Amount,10.44>\\ \; & <ID,102><NS2-3,depositMoneyResponse><return,3465> \end{array}$$

For example, we select cluster 2 to generate the value for the field with the key return to follow the steps. We go through the numeric module step by step to show its evaluation on this data set and its limitations along the way.

#### 5.1.8. Request Decomposition and Evidence Collection

After the numeric keys in each RT and the centroid interaction from the corresponding cluster are identified, each cluster centroid is used in order to develop a model that expresses how to generate a numeric value in the RT of the cluster. Given that the CSV can be applied to stateful and stateless protocols, other influential messages may exist apart from the immediate request that have an influence on the numeric value of a field. Therefore, we have to incorporate a long trace of past messages to obtain as much evidence as possible.

Deciding which interactions to include in the historic trace requires criteria. For this purpose, we introduce the concept of searchable fields. Among the fields $m_{q_{c}}=<k_{1},v_{c_{1}}><k_{2},v_{c_{2}}>\ldots <k_{n},v_{c_{n}}>$ of the centroid request, the fields with categorical values lend themselves as criteria. The identification of the type of a field is nontrivial and potential future research. At present, a user is required to pre-select categorical fields. In the banking example, in the cluster containing interactions with request message type equals to depositMoney and centroid request $m_{c}=<ID,101><NS2,depositMoney><AccountID,244-652-34><Amount,10.44>$, the categorical fields are <NS2,depositMoney><AccountID,244−652−34>. The CSV applies all combinations of the searchable fields to gather different collections of messages that we define as evidence collections because the importance and relevance of each searchable field are unknown. The combination of searchable fields used to create the evidence collection is called a filter. Each evidence collection may consist of one or several message types, depending on the filter that was used. This component treats any interaction in the history as equally important in the search process. Evidence collections in the CSV are conducted by the following rules:

1. A number of evidence collections are gathered from the recorded messages on the basis of the combinations of searchable fields in each centroid request message. The combinations lead to $2^{r}-1$ of non-empty subsets of searchable fields set, where *r* is the number of searchable fields in the centroid request. In the banking example, with the two searchable fields <NS2,depositMoney><AccountID,244−652−34>, three different combinations may exist: Filter 1 is {<NS2,depositMoney>}, Filter 2 is {<AccountID,244−652−34>}, and Filter 3 is {<NS2,depositMoney>,<AccountID,244−652−34>};
2. Interactions that have one or more target fields in their responses are collected and added as an additional evidence collection. Each numeric key in each RT is considered to be a target field that the system is expected to generate a correct value for. In the same cluster of the banking data set, Filter 4 finds interactions with {<return,?>} in their responses as another collection.
3. Combining rules 1 and 2 results in additional numbers of collections. In the case of the example cluster of the banking data set, interactions that have *return* key in their response messages are also filtered using Filters 1–3, thereby leading to additional filters, Filter 5={<return,?>,<NS2,depositMoney>}, Filter6= {<return,?>, <AccountID,244−652−34>} and Filter7={<return,?>, <NS2,depositMoney>, <AccountID,244−652−34>}, which retrieve additional evidence collections.

The use of different filters in an evidence collection may lead to identical collections. After creating the evidence collections by using all of the filters, duplicated collections are identified and removed. Accordingly, different evidence collections may contain keys with numeric values from relevant messages that provide the data that the system can use to learn the model for deriving the target field. In the banking data set, two in seven collections that were created by the seven filters were removed as duplicates. The result of evidence collection step for the above example is shown in Table 1.

#### 5.1.9. Mapping

The mapping component finds responses that contain target fields of the centroid request type. This component maps each target in each collection to its earlier evidence collections. Based on the IDs that were assigned to messages during the pre-processing, the evidence messages for each target message can be defined as all messages in that collection that have an ID greater or equal to one and smaller than the ID of the object response containing the target field. Next, each evidence message keys are tagged with their message types. For example, in the example message, we have *m*=<ID−depositMoney,101><NS2−depositMoney,depositMoney><AccountID−depositMoney,244−652−34><Amount−depositMoney,10.44>. Later, all of the non-numeric fields of the messages are removed from the collections. In the above example, after removing categorical data and non-numeric fields, the remaining fields are m=<ID−depositMoney,101><Amount−depositMoney,10.4>. Finally, the evidence messages for each target message are sorted on the basis of their message IDs in an ascending order.

#### 5.1.10. Building Models

In this step, all remaining evidence fields from the previous step are considered to be input for the target fields in order to identify the possible relationships between evidence and target fields. To this end, 10-fold cross-validation for the evaluation [30] is used. The data set is randomly split into ten separate sets, and one of them is selected for the evaluation. The remaining nine sets are used for training.

In stateful protocols, the value of a field can depend on a history of a variable number of messages. The last *m* consecutive messages immediately before the target message are selected to incorporate all of the necessary messages from the evidence collection in calculating the target values. The goal is to ensure that the model only includes the minimum of prior messages that are needed to derive the target value. The model is built starting from m=1, which means the last message before the target message is selected as input of the model. m is incremented until the target value is correctly derived for all training cases. Every increment goes back one message further into the past.

The machine learning models can be chosen on the basis of the problem complexity if any prior knowledge of it exists or using a rich predefined set of models that can cover most of the possible relationships, such as linear, polynomial, and exponential. History length *m* is increased whenever the selection of potential relationships is not successful in achieving the desired accuracy. This procedure is repeated and continued until the required accuracy has been achieved or until the achieved accuracy is decreased while increasing the length of history.

This process is carried out in Weka [31] for our evaluation. We used the MultiScheme class that selects one classifier among several and applies a performance measure (mean squared error). In our data sets, we have included the SMOReg classifier with polykernel for covering polynomial dependencies, MultiSearch with LinearRegression for linear relationships and ZeroR as a baseline classifier. The other classifiers can be added on the basis of the complexity of the data sets. In service virtualisation problem, we may have different applications that may require different level of accuracies. In some applications, the accuracy is critical; however, in other scenarios the accuracy of generated virtual service may not be as important. The baseline classifier can be selected based on the application.

Given that model building uses data mining methods, the number of instances in each evidence collection should not be smaller than the required number of instances for the data mining methods. This number varies from method-to-method and it depends on the number of features. The number of message types that the system is required to virtualise responses also influences the number of traces required to learn the models and the required length of traces *m*.

#### 5.1.11. Model and Filter Selection

As a result of the previous step, we have a filter used to create the evidence collection in each evidence collection. We also have *m* models; each one is built on the basis of the last 1,…,m consecutive messages. Among all *m* models in each collection, the model with the best performance, measured in terms of the mean squared error, and the number *m* of past messages that produced the best outcome using the model, are selected as the outcome of the learning process. The filter that is used in order to create the collection with the optimal accuracy is chosen as the selected filter.

### 5.2. Playback Mode

In offline mode, the model and its filter for generating each numeric field in the RT were identified. In the playback mode, the system accepts live requests and generates the response messages and values of their numeric fields.

#### Response Generation

When a live request is received, its message type and other relevant fields are used in order to select the RT, the model and the filter that creates the relevant evidence collection for generating the response message. The numeric fields of the chosen RT are generated while using the corresponding models. The live request and response created are paired, numbered, and added as an interaction to update the pre-processed traces. This step is necessary in order to ensure that the subsequent requests that rely on the history of virtualised responses can be correctly answered.

## 6. Data Set Descriptions

In this section, each data set used in our evaluation is introduced in detail. First, a banking data set will be explained. This data set was generated to be as realistic as possible and it contains the transactions between an Automated Teller Machine (ATM) and its banking server. Subsequently, a calculator is the second generated data set that simulates the network transactions between a calculator API and its client. Finally, an LDAP data set, recorded from a real scenario, will be explained. This data set contains the transactions between the LDAP client and its server.

### 6.1. Banking Data Set

**Figure 6:**
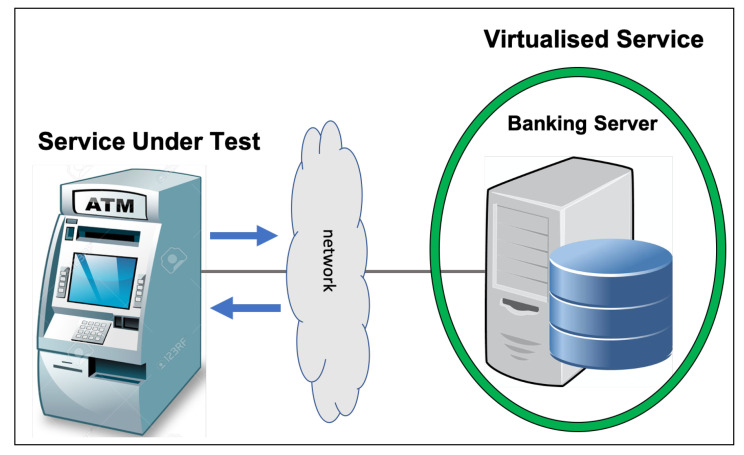
Banking scenario.

Figure 6 shows that the banking data set contains transactions between an ATM and a banking server. To test the ATM service, the banking server has to be virtualised in order to ensure that it can produce authentic server responses. The ATM is assumed to communicate with the server, receive return values, and account information after different transactions, such as requests for account information, deposits, and withdrawals. In the banking data set, six types of request messages are present, namely getNewToken, deleteToken, getTransactions, depositMoney, withdrawMoney, and getAccount.

Two of these message types, namely, depositMoney and withdrawMoney, require numeric fields in their responses. The banking server has to return the exact value of each account’s balance after completing the operation in each request. CSV has to learn to add values and subtract in the deposit and withdrawal interactions. Moreover, CSV has to find the previous balance from the trace of prior interactions as a base value. Here, Listing 8 shows banking data set samples.

**Figure 7:**
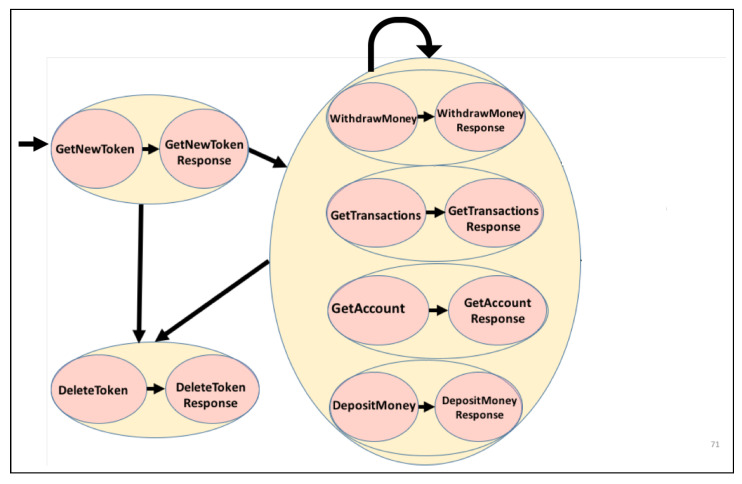
Banking data set control flow.

The banking data set has 300 traces, containing a total of 1126 interactions that pertain to 10 different account IDs. The length of each trace’s ranges from three to eight messages. getNewToken and deleteToken indicate the start and end of each trace; therefore, they are repeated in each trace. Zero to eight, which are the [0–8] number of four types of messages, namely, getTransactions, depositMoney, withdrawMoney, getAccount, can appear. The banking data set control flow is shown in Figure 7.

**Figure 8:**
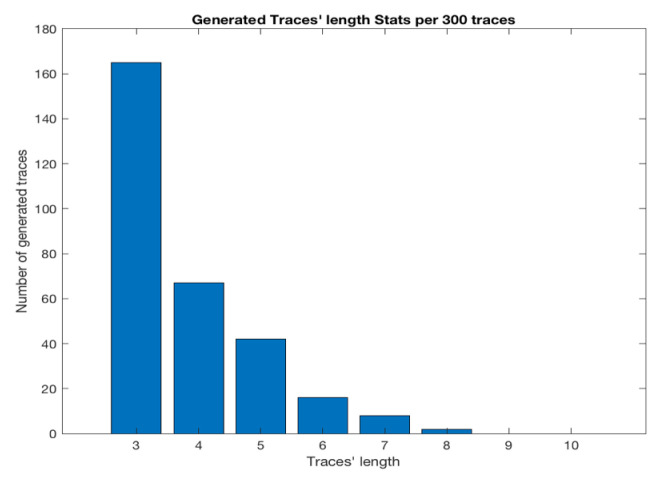
Generated banking traces’ length per 300 traces.

Two factors are important and they need to be defined to have the data set that follows the ATM users’ behaviours. The first factor is the number of traces with different lengths. In reality, the number of transactions requested by ATM users between each log in, which is specified by getNewToken and log out by deleteToken, tends to be short (e.g., one or two transactions than seven or eight transactions). The beta distribution [32] was used in order to determine the number of traces with different lengths by using parameters α and β equal to 0.7 and 3.3, respectively, to efficiently match the generated data set to the tendency of ATM’s users in a real scenario (Figure 8). The second factor is the type of messages that appear in each trace. The uniform random distribution was used to generate each message type among four possible types in traces. Specifically, each message type has an equal probability of being selected in each trace.

**Listing 8:**
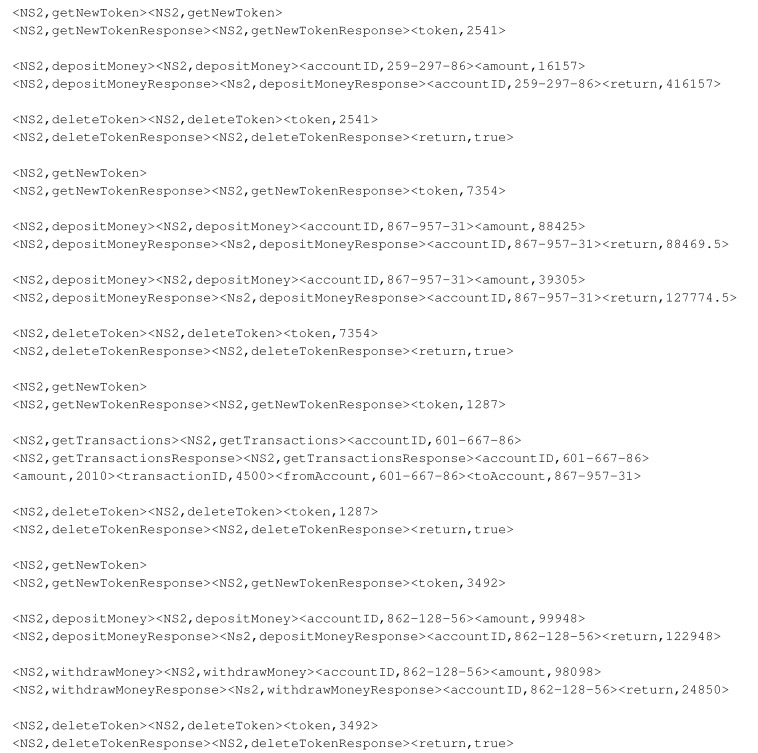
Banking data set samples

The data set must contain a “sufficient” number of traces with representative numbers and combinations of types of interactions to ensure that the CSV learns how to accurately predict numeric fields. In future work, we will investigate the number of different types of interactions that the method will need for the approach to effectively work.

### 6.2. Calculator Data Set

**Figure 9:**
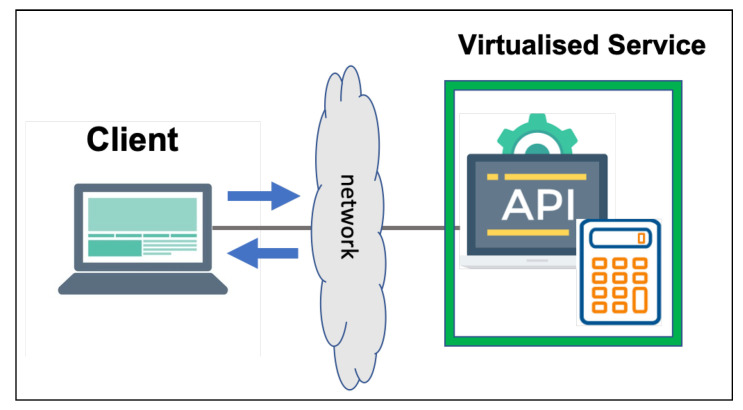
Calculator scenario.

This data set simulates the interactions between a calculator and its client (Figure 9). Such a data set contains 5000 interactions with seven different request message types, namely, clear, add, addResult, sub, subResult, multiply, and multiplyResult. Each type is followed by its respective unique response type. The message types are roughly equally represented in the data set. The calculator data set consists of simple non-interleaving interactions. The clear request is always followed by a response with a result of zero.

The add, sub, and multiply messages have two operands in their requests and they are followed by their response messages that contain the result of their operation on two operands. The requests of the addResult, subResult, and multiplyResult message types have one operand in their requests, which is followed by their result. These result fields of messages with a single operand naturally depend on the value in the preceding response message and the current request message. Here, Listing 9 presents some messages from this data set.

**Listing 9:**
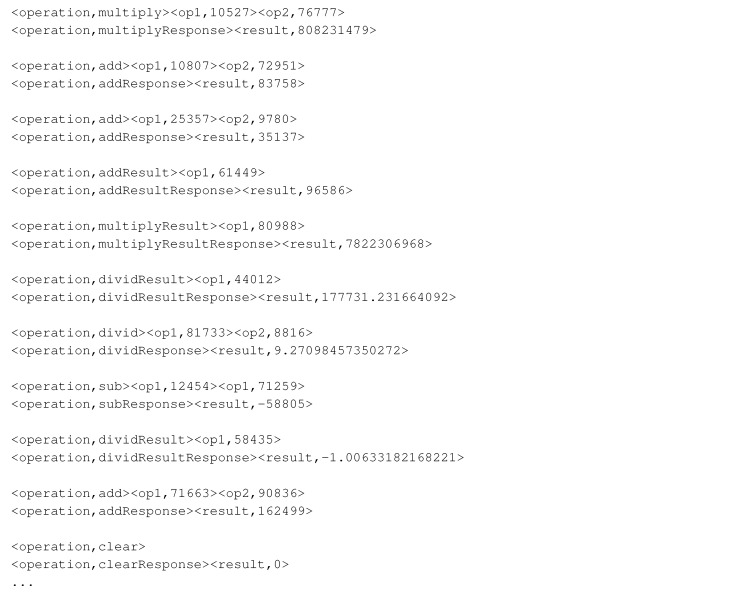
Calculator data set samples

### 6.3. Ldap Data Set

The Lightweight Directory Access Protocol is an industry standard protocol for accessing and managing directory information service over the Internet [25,26]. The LDAP data set contains 100 traces and 2183 interactions between the LDAP client and the server. This data set has been recorded from a real client–server exchange and it contains eight different message types. Here, Listing 10 illustrates some messages from this data set.

Each message in the LDAP protocol contains a field with the key messageID, which specifies the order of message in each trace. Each trace starts with a bindRequest request that has a messageID of one, which is incremented by one in each subsequent request throughout the trace that ends with an unbindRequest request type. Each LDAP request message in the data set has, at most, one response message with the same messageID as its request. The CSV is used to generate the value for messageID, which is the only non-categorical numeric value in the response messages.

**Listing 10:**
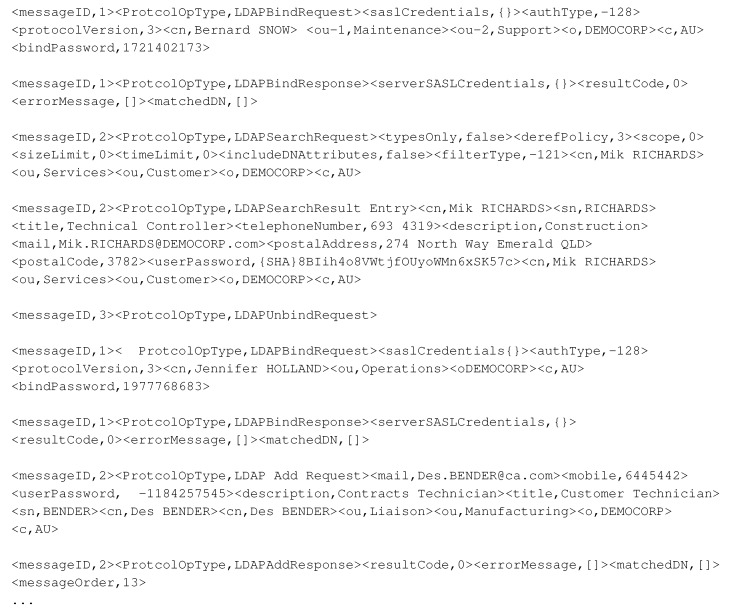
LDAP data set samples

## 7. Evaluation

This section contains the result of testing CSV on three data sets. CSV was implemented in Matlab. In the banking scenario, the ATM as the SUT sends either a withdrawMoney or depositMoney request message with the specific amount that is related to one of the existing account IDs as part of its interaction. The virtualised service responds to the request from the ATM. A model with m=2 consecutive messages was correctly selected as the optimal model. Filter 2={<AccountID,V>} was correctly identified as the appropriate filter. Given that deposits and withdrawals are the only interactions that contain numeric fields in the responses, the experiments were based on these types of interactions. Table 2 shows that CSV can accurately derive the target field for all deposits and withdrawals of the test set. Table 3 shows the numbers of test cases used and correctly virtualised responses.

In the case of the calculator data set, CSV generates accurate values for the result fields of all seven different message types. The result value in the response of the clear request was identified as a constant by the component that creates the RT. The model with linear complexity achieved the optimal accuracy and it was selected for the add, addResult, sub and subResult request types. The SMOReg function with a polykernel model optimally performed in terms of accuracy for multiply and multiplyResult. In all cases, the interactions with {<result,V>} in the responses were the evidence collections, which achieved the best accuracy. Therefore, the corresponding filter was chosen as a basis for the choice of evidence. In operations with two operands, such as multiply, the number of relevant messages in the trace is correctly identified as m=1, because all of the information that the model needs is in the request. In single-operand cases, the system correctly chooses m=2.

In the LDAP data set, the ‘creating RT’ component attempts to find an RT by using simple intra-message and inter-message (request—response) dependencies; therefore, it detects the equivalence between request messageID and response messageID. Given the messageID values always equal to one that in the bindRequest, it identifies the messageID in the bindResponse as the constant in the RT. The numeric field of messageID in the other RTs are identified as equal to the same key in their corresponding request types as the result of the ‘creating RT’ component.

Table 2 compares the results of CSV with those that were achieved by Opaque SV when generating non-categorical numeric data in the banking, calculator and LDAP data sets. In this work, the accuracy is defined as the ratio of correctly generated non-categorical numeric values in the response messages to the number of all non-categorical numeric values in all of the responses that were generated. CSV accurately generates all numeric fields for all three data sets with error rate (residuals) of zero. Meanwhile, Opaque SV can generate 100% accurate results in the case of LDAP, which only has equality relationships; it can successfully generate responses to the clear request type of the calculator data set, which requires a constant value of zero. Opaque SV was not designed to create values for numeric fields; accordingly, it failed in all cases of the banking data set.

Table 3 shows the number of correctly generated response values. The creation of the responses included generating the template and response values. Although the CSV can cover majority of the fields, the CSV cannot predict other fields, such as encrypted fields, hash fields, and tokens, which were excluded from our evaluations.

In the end, Table 4 compares the related SV and the proposed CSV solutions on four different benchmarks. As the table shows, CSV presents an automatic solution that gains high accuracy in generating numeric fields. Besides, the CSV learns from the recorded interactions in a short time while others fall short in one or more benchmarks. Even though both Kaluta and Commercial SVs gain high accuracy, they require service specifications, long time and massive human effort to build the virtualised services manually.

## 8. Threats To Validity

Because the cognitive SV uses data mining methods in the building models step, the number of instances in each evidence collection should not be smaller than the required data mining methods. The number depends on the method and the number of features. The required number of training instances increases with the increase in the number of relevant message types.

The number of training instances depends on the scenario. In some cases, several historic messages may have to be considered in order to derive an accurate value for a response field. For example, in the banking data set, the last message could be a message with type depositMoney or withdrawMoney. Given that each type has two numeric fields as features, namely, amount and result, four features would be necessary for the model to learn the relationship with the target. Consequently, two consecutive messages contain eight numeric fields that need to be examined in order to discover the relationship between them and the target.

Furthermore, the data set needs to be rich enough to contain all possible combinations of influential messages in the training data set that may appear later in the test data set. The number of combinations of influential message types in the training data set also has to be large enough for the model to learn the relationship before testing.

The system perceives its state, because it is reflected in the recorded transactions. The system has to be presented with a full and current set of relevant messages. Some constraints are observed on the training and test data set of the system. Given that the CSV learns a model per request message type, the training data set needs to cover all of the message types that will be received on the playback mode as the test set. The current implementation of CSV cannot handle unseen request types in the playback mode. In scenarios that need previous states for calculating the current numeric values, the messages that are received on playback mode need to have some relevant interactions in the training data to establish their states.

## 9. Conclusions and Future Work

In this study, CSV, which is a novel approach for accurately generating the values of numeric fields in response messages, has been proposed. CSV finds the messages and fields that are relevant in generating the numeric response for each type of request message and infer the logic and semantic behind message types that affect the numeric response fields. The numeric response fields are then combined in order to generate the closest possible value to the expected response value for this field. We evaluated the solution on three use case scenarios, each with different levels of complexity and mathematical relationships that had to be identified by the approach. In the test data sets, CSV achieved an accuracy of 100% for generating values for numeric fields. The banking data set contains a linear mathematical dependency that has been successfully discovered. The calculator data set contains linear and polynomial dependencies that the CSV successfully identified. The LDAP data set features a simple equivalency dependency that only required the step of RT generation.

In spite of the success demonstrated at deriving numeric values for the response messages, a number of open problems remain. In future work, we plan to address the following three key issues: (i) expanding the solution approach to a framework and making it more general and efficient to ensure that many use case scenarios can be covered, (ii) extending the approach on how learned models are constructed to ensure that it can deal with ‘incomplete’ training sets, and (iii) expanding existing work to create reliable predictors for non-numeric fields. The approach also needs to be validated on further data sets that contain dependencies between numeric fields that go beyond the dependencies of the data sets that were used in this study. Finally, we need to further investigate the scale and ‘diversity’ requirements for training data sets to ensure that accurate models can be mined.

## Figures and Tables

**Table 1:** Evidence collection result for the banking data set.

Collections	Filter	Type 1	Type 2	Type 3	Type 4	Instance Number
Collection 1	NS2-1: depositMoney	depositMoney	-	-	-	232
Collection 2	accountID:244-652-34, NS2-1:depositMoney	depositMoney	-	-	-	31
Collection 3	accountID 244-652-34	getAccount	getTransaction	withdrawMoney	depositMoney	31
Collection 4	Interactions with *return* field in response message	withdrawMoney	depositMoney	-	-	232
Collection 5	Interactions with *return* field in response message, accountID:244-652-34	withdrawMoney	depositMoney	-	-	31

**Table 2:** Comparison between CSV and Opaque SV accuracies on all three data sets.

	Data Set	Banking	Calculator	LDAP
Method				
Cognitive SV		100%	100%	100%
Opaque SV		0%	13.5%	100%

**Table 3:** Number of numeric fields in all message types in each protocol and the number of correctly generated responses by CSV.

	Data Set	Banking	Calculator	LDAP
Stats				
Number of correctly generated response values		10	14	26
Number of all numeric values		10	14	26

**Table 4:** Comparison between different SV methods on generating non-categorical numeric fields.

	Methods	Kaluta [17]	Commercial SV [18,19,20]	Opaque SV [15]	LSTM-Based Method [21]	CSV
Benchmarks						
Accuracy		High	High	Very low	Low	High
Automatic/Manual development		Manual development	Manual development	Mostly automatic	Mostly automatic	Mostly automatic
Requirement		Service specification	Service specification	Recorded interactions	Recorded interactions	Recorded interactions
Development/Training time		Long	Long	Short	Long	Short
